# Exploring the mechanism of live streaming e-commerce anchors’ language appeals on users’ purchase intention

**DOI:** 10.3389/fpsyg.2023.1109092

**Published:** 2023-03-09

**Authors:** Erwei Ma, Jiaojiao Liu, Kai Li

**Affiliations:** ^1^School of Journalism and Communication, Zhengzhou University, Zhengzhou, China; ^2^School of Journalism and Communication, Guangxi University, Nanning, China

**Keywords:** live streaming e-commerce, language appeals, self-referencing, self-brand congruity, purchase intention

## Abstract

**Introduction:**

Live streaming e-commerce is an important way for consumers to shop nowadays. Anchors, as salesperson in live streaming e-commerce, greatly affect the sales of the broadcast room. This paper studies the influence mechanism of anchors’ language appeals, rational appeal, and emotional appeal on users’ purchase intention. This study establishes a research framework which based on stimulus-organism-response (SOR) theory, and constructs a model to reflect the relationship between anchors language appeals, self-referencing, self-brand congruity, and purchase intention.

**Methods:**

Survey using a convenience sample (N = 509) was conducted on Chinese mainland netizens through WJX platform (October 17-23, 2022) to obtain data. The partial least square structural equation modeling (PLS-SEM) method was used for data analysis.

**Results:**

The study found that anchors’ language appeals was positively correlated with self-referencing and self-brand congruity, and there is a positive correlation between self-referencing, self-brand congruity, and purchase intention. Self-referencing and self-brand congruity play a mediating effect between anchors language appeals and purchase intention.

**Discussion:**

This study advances the literature on live streaming e-commerce research and SOR and provides practical implications to influence the strategy of the e-commerce anchors.

## 1. Introduction

Live streaming e-commerce emerged in China in 2016, and several e-commerce platforms tested the live streaming e-commerce model (Fan et al., 2022). In 2016, Amazon was the first to launch a style code live show, where netizens watch a live stream and can place a purchase directly through a link below the video. Facebook also introduced a live e-commerce service in 2018. With this service, merchants start a live stream on the platform, users take a screenshot of their selected products, submit it to the merchants, and the merchant’s replies with payment instructions *via* messenger. Data show that in 2019 the US live e-commerce scale was less than US$1 billion (Coresight Research, 2020), in just 2 years it soared to US$11 billion in 2021 and is expected to exceed US$25 billion in 2023 (Coresight Research, 2022), which is a very rapid growth. Since 2018, the development of China’s live-streaming e-commerce industry has become an industry trend, attracting a large number of funds and institutions to enter, with a surge in practitioners and market size. According to data released by the Department of E-Commerce of the Ministry of Commerce of the People’s Republic of China, the overall market size of live e-commerce in China will reach 1.05 trillion yuan in 2020 (Ministry of Commerce of the People's Republic of China, 2020), far exceeding the $6 billion of the United States, the world’s largest consumer market (Paper News, 2022). With the growth of live e-commerce, anchors engaged in live-streaming with goods are rapidly emerging, with celebrities, influencers, and government officials of all stripes joining the live-streaming bandwagon. During Amazon’s Members’ Day 11–13 July 2022, comedian Kevin Hart, Australian model Miranda Kerr, and actress Kyle Richards, among others, joined Amazon’s live-streaming bandwagon anchors (NetEase News, 2022). An e-commerce anchor is a person who interacts with users and recommends products in a live stream (Sun et al., 2019; Park and Lin, 2020). With their unique personal charisma and professional knowledge, e-commerce anchors recommend products to users in various languages, talk about their experiences in trying or using products, and answer their pop-up questions in the live stream, which not only enhance the consumption atmosphere in the live stream, but also strengthen the close connection with users, alleviate their worries when shopping, and promote them to click to place a purchase. As an important link between consumers and products, e-commerce anchors have become a key influence on live streaming traffic and transaction volume, and their value is highly valued.

The strong marketing influence of e-commerce anchors on consumers has attracted scholars’ research attention. Some scholars have studied the impact of anchor features on users’ purchase intention. The more authoritative anchor identity is, the more it will stimulate consumers’ purchase intention (Liu F. et al., 2020). The credibility and professionalism of anchors will affect consumers’ purchase intention (Han and Xu, 2020). Some scholars have studied the influence of anchors’ communication strategies on consumption intention. For example, the more similarity of communication styles between anchors and users, the higher the users’ purchase intention (Wu et al., 2020). The interactivity of anchor language will also affect consumers’ purchase intention (Sun, 2022). According to the study of Hu and Chaudhry (2020), anchors provide consumers with personalized recommendations, guidance, and services by means of in-line dialogue and barrage questioning, which has a positive impact on consumers’ purchase intention. Anchors use voice, expression, or movement skills to answer consumers’ questions, which can promote consumers to make purchasing decisions (Cai and Wohn, 2019).

Scholars have identified language appeals as an important factor influencing consumer behavior (Gong et al., 2021). Research in this area has also attracted the attention of researchers, focusing on two main areas. (1) The influence of linguistic appeals in advertising on consumer behavior. Some scholars focus on research on the type of advertising and the use of appeal strategies, e.g., some scholars focus on different types of advertising (Kim et al., 2019), while others have studied the use and effects of appeal strategies in the sale of different types of products (utility, hedonic, and technological; Kronrod and Danziger, 2013; Gahlot Sarkar et al., 2019; Motoki et al., 2019; Srivastava and Dorsch, 2020). Some scholars have focused on the mechanisms of appeal strategies on purchase behavior, such as the effect of celebrity endorsement on purchase behavior (Wang et al., 2013), the effect of inaccessible strategies such as emotional appeal, ability appeal, functional appeal, and experience on purchase behavior (Zarantonello et al., 2013; Kazakova et al., 2016; Kim et al., 2019). (2) The effect of language appeals of anchors on consumer behavior in TV shopping. Scholars study found that Language appeals is an important communication strategy for anchors. The language appeals of anchors is a vital factor in promoting consumers’ purchase intention (Luna and Peracchio, 2005; Bishop and Peterson, 2010). Some researchers concentrate on the impact of communicators on consumers’ purchasing decisions, including celebrities, specialists, and professional anchors (Ma and Jongchang, 2019; Zafar et al., 2020; Sun, 2022). Some researchers concentrate on understanding the mechanisms through which various appeal methods, including logical appeal, emotional appeal, and personalized appeal, affect consumers’ perceptions of brands and their propensity to make purchases (Shih, 2011; Siddiqui and Nabeel, 2014; Jayathunga and Kumara, 2016; Luo et al., 2021). Webcasting has developed into a significant means of influence because of the growth of live online commerce. However, there are few researches on the influence mechanism of webcasting e-commerce about anchors’ language appeals and users’ purchase intention. Research on this relationship is valuable because it helps to explain how the language appeals of anchors contributes to the purchase intention of users. At the same time, the research results are helpful to promote the sales performance of live shopping. Therefore, the first research question of this paper is put forward: What is the influence mechanism of anchors’ language appeals on users’ purchase intention?

This study contributes in four ways: firstly, it extends current research on Internet consumer behavior by exploring how anchors’ language appeals affect users’ purchase intention. Secondly, it helps to deepen our understanding of the mechanisms by which anchors’ language appeals influence users’ purchase intention. Thirdly, the findings of the study can serve the development of promotional strategies for live marketing campaigns; fourthly, broadens the applicable scenarios of the stimulus-organism-response (SOR) theoretical framework, particularly in the context of China.

## 2. Theoretical framework and hypotheses

### 2.1. Theoretical basis

The stimulus-organism-response model was proposed by Mehrabian and Russell (1974) as a framework for explaining the effects of external environmental stimuli on individuals’ cognition, emotion, and behavior. The SOR model is widely used to predict and explain consumer behavior in online marketing environments. Li et al. (2022) explored the effect of social presence in live streaming on customer impulse buying based on the stimulus–organism–response framework. Hu and Chaudhry (2020), used the SOR model to study how relationships (social and structural bonds) in live e-commerce enhance consumer engagement. The structural integrity of SOR model and its classification of the process of the influence of information stimulus on human behavior have been widely recognized by researchers. Therefore, this study uses the SOR model as the theoretical basis, anchors’ language appeals as the stimulus, self-referencing and self-brand congruity as the organism state, Anchors’ language appeals as the stimulus, self-referencing and self-brand congruity as the organism state, and purchase intention as the behavioral response.

#### 2.1.1. Stimulus factors-anchors’ language appeals

“Stimulus” is a “trigger” (Mehrabian and Russell, 1974) that causes a change in the internal or external state of an individual, which can be a source of information, message content, etc. According to Turley and Kelley (1997), language appeals to product promotion can be divided into rational and emotional appeals; Pang and Lee (2004) showed that rational and emotional appeals are two types of appeals that can significantly influence consumption intention. Based on this, this study classifies anchors’ language appeals into rational appeal and emotional appeal. Rational appeal mainly changes consumers’ psychological perceptions and purchase behavior by conveying factual information, such as product recommendations in terms of price, quality, and composition (Resnik and Stern, 1977). Affective appeal influences consumers’ psychological perception and purchase behavior by stimulating consumers’ emotions toward the brand and even toward the salesperson, such as product recommendation in terms of humorous appeal and nostalgic appeal (De Pelsmacker and Geuens, 1997).

#### 2.1.2. Organism state: Self-referencing and self-brand congruity

“Organism” refers to the emotional and cognitive mediated states that arise when an individual interacts with external stimuli (Mehrabian and Russell, 1974).

Self-referencing refers to an individual comparing external information with self-relevant information stored in memory (Debevec and Romeo, 1992). Research has found that there is a self-referential effect on consumers’ live shopping behavior, i.e., consumers compare the anchor with their self-characteristics (image, beliefs, personality, abilities, etc.), and the stronger the identification, the stronger their willingness to purchase (Hu et al., 2017). Study has found that when consumers receive information about products, they associate the information with their personal experiences, triggering the phenomenon of self-referencing，the outcome of treatment can affect their purchase intention (Burnkrant and Unnava, 1995; Martin et al., 2004). Research indicates that self-referencing is an important factor influencing consumers’ purchase decisions (Chang, 2011; Yoon and Park, 2012).

Consumer buying behavior is not just about satisfying physical needs, but also about satisfying psychological desires. Consumers tend to buy brands that help express and shape their self-image (Levy, 1959). Gardner and Levy (1955) refer to this phenomenon as self-brand congruity. The similarity and match between self-image and brand image. Study found that consumers make congruent judgments after acquiring information about products, and when consumers produce stronger congruence, they show stronger willingness to purchase (Roy and Rabbanee, 2015). When users watch a live broadcast, they combine the brand image perceived through the linguistic description of the anchor with their own image, and the stronger the congruence between the two, the stronger their purchase intention (Islam et al., 2018). Many studies have pointed out that self-congruity (self-consistency) is an important factor that influences consumers to choose and purchase products (Peters and Leshner, 2013).

Based on the above discussion, self-referencing and self-brand congruity are both emotional and cognitive responses that occur when individuals interact with external stimuli (Sirgy, 1982; Bellezza, 1984). In this study, self-referencing and self-brand congruity were used as mediating factors.

#### 2.1.3. Consumer behavior response: Purchase intention

The user’s purchasing intention is referred to as the behavioral reaction in this study. Purchase intention is the subjective probability or potential that a consumer will purchase a specific good (Dodds et al., 1991). Purchase intention, according to Søndergaard et al. (2005), can be viewed as the irrational propensity of consumers to select particular goods, which develops during the process of product or service cognition prompted by external impacts.

### 2.2. Hypotheses

#### 2.2.1. Relationship between anchors’ language appeals and self-referencing

E-commerce anchors recommend products to consumers through language appeals (Sun et al., 2019). In this study, anchor appeals are divided into rational appeals and emotional appeals. It has been noted that both rational and emotional appeal evoke consumers’ memories of their own experiences and trigger self-referential behaviors (Wang et al., 2013; de Graaf, 2022). On the one hand, rational appeal is used to motivate consumers by providing factual information (Lee et al., 2020). Emotional appeal, on the other hand, is used to arouse consumers’ memories by stimulating their emotional responses (Lee et al., 2020). Anchors’ language demands for products will increase users’ self-information cognition and stimulate users’ self-reference to a certain extent. As a result, this paper proposes hypothesis 1.

*H1a*: Anchors’ rational appeal has a positive influence on self-referencing.

*H1b*: Anchors’ emotional appeal has a positive influence on self-referencing.

#### 2.2.2. Relationship between anchors’ language appeals and self-brand congruity

Past research has found that consumers matched product’s utilitarian attributes with their own ideal attributes through rational appeal, and matched product’s value express attributes with their own self-concept through emotional appeal, creating self-brand congruity (Johar and Sirgy, 1991). When customers receive information, they will relate it to their self-image and have a tendency to choose brands that are consistent with their self-image (Heath and Scott, 1998). When the values conveyed by the brand are related to the values of consumers, consumers will have a positive attitude toward the brand (Michel et al., 2022). Anchors’ language appeal to the brand will arouse users to compare the brand image with their self-image. If the two are consistent, they will increase their purchase intention. Thus, this paper proposes hypothesis 2:

*H2a*: The rational appeal of the anchor has a positive impact on self-brand congruity.

*H2b*: The emotional appeal of the anchor has a positive impact on self-brand congruity.

#### 2.2.3. The influence of self-referencing on purchase intention

Self-referencing impacts customers’ buying intentions (Debevec and Iyer, 1988; Wang et al., 2013). Consumers buy products in order to maintain or improve the self-image they pursue. The self-reference effect causes consumers to compare their self-image with the product image. A high level of self-referencing will provide more recognition and a larger desire to make a purchase (Wang et al., 2013; Yim et al., 2021). When users watch live broadcasts, they will empathize with and recognize the language demands of anchors, and trigger the self-reference effect, which will strengthen the psychological connection between users and anchors and further affect the purchase intention. Thus, this paper proposes hypothesis 3:

*H3*: Self-referencing influences purchase intention in a favorable way.

#### 2.2.4. The influence of self-brand congruity on purchase intention

Previous studies have pointed out that self-brand congruity has an important impact on consumers’ purchasing behavior (Ekinci and Riley, 2003; Rabbanee et al., 2020). It is found that the higher the self-brand consistency of consumers, the more positive their attitude toward the product, the more likely they are to have the purchase intention (Liu C. et al., 2020; Wijnands and Gill, 2020). Customers are more likely to make purchases when their self-image is extremely aligned with that of the brand (Phua and Kim, 2018; Chen et al., 2021). Through rational and emotional appeals of anchor language, users connect self-concept with brand-image to improve their purchase intention. Hence, this paper proposes hypothesis 4:

*H4*: Self-brand congruity has a positive impact on purchase intention.

#### 2.2.5. The influence of self-referencing on self-brand congruity

Based on previous studies, the more self-referential consumers perceive, the more consistent their selves are with the brand, and the more likely they are to demonstrate a stronger sense of engagement, loyalty, and identity (Mehta, 1999). Through self-referencing, consumers compare information with themselves to promote the congruity between self-image and brand image. It has been found that consumers’ self-referential behavior helps to facilitate consumers’ association with brands and increase self-brand congruence (Wang et al., 2013). This paper argues that self-referencing triggered by anchors’ verbal appeals affects users’ self-brand congruence. Thus, this paper proposes hypothesis 5:

*H5*: Self-referencing has a positive effect on self-brand congruity.

#### 2.2.6. The mediating role of self-referencing between language appeals and purchase intention

Individuals’ perceptions of verbal and visual stimuli can induce their self-referencing behaviors and then influence their attitudes and intentions (Debevec and Romeo, 1992). The appeals by communicators can stimulate consumers’ self-referencing behaviors, thus influencing consumers’ attitudes toward products (Yoon and Park, 2012). Further research has found that both verbal and non-verbal appeals can stimulate self-referential behavior, which in turn influences consumers’ purchase intentions (Wang et al., 2013). The self-referencing effect is mediated by the fact that users are influenced by the verbal appeal of the anchor, which stimulates self-referencing and increases their perception of their self-image, and when their perception of their self-image matches the product recommended by the anchor, they increase their purchase intention. Thus, this paper proposes hypothesis 6.

*H6a*: Self-referencing mediates between rational appeal and purchase intentions.

*H6b*: Self-referencing mediates between emotional appeal and purchase intentions.

#### 2.2.7. The mediating role of self-brand congruity between language appeals and purchase intention

Research has found that people will consider the degree of consistency between a product’s brand image and their own image when screening the product, and the higher this consistency, the higher people’s willingness to purchase the product (Yang, 2018; Chen et al., 2021). When consumers are exposed to brand information, they develop brand image perceptions and tend to purchase the brand if they perceive that their self-image and the image portrayed by the brand converge, or if they believe that the image portrayed by the brand can satisfy their self-image (Liu C. et al., 2020; Wijnands and Gill, 2020). In a study on brand filter conditions, self-brand congruence between brand filter conditions (self-endorsing vs. other-endorsing) and brand attitudes, purchase intention Yang (2018). Based on the existing studies, this paper further explores whether self-brand congruence plays a mediating effect between anchor language appeals and purchase intention. Thus, this paper proposes hypothesis 7.

*H7a*: Self-brand congruity plays a mediating role between rational appeal and purchase intention.

*H7b*: Self-brand congruity plays a mediating role between emotional appeal and purchase intention.

#### 2.2.8. The chain mediating role of self-referencing and self-brand congruity

As mentioned in the previous section, the anchor’s rational and emotional appeal act as stimuli to trigger consumers’ self-referencing and self-brand congruity, which in turn influence consumers’ purchase intentions. Meanwhile, self-referencing has an impact on the relationship between anchors’ language appeals and consumers’ self-brand congruity (Mehta, 1999). Research suggests that self-referencing encourages consumers to connect with brands and increases self-brand congruence, which in turn influences consumers’ purchase intentions (Wang et al., 2013). A study by Phua and Kim (2018) also found that self-brand congruence interacts with self-referencing and perceived humor to influence consumers’ brand attitudes and purchase intentions. Users perceive brand messages through anchor language appeals, and if users’ self-reference increases, self-concept, and brand image are more likely to be congruent, which in turn increases purchase intentions. Thus, this paper proposes hypothesis 8:

*H8a*: Self-referencing and self-brand congruity play a chain mediating role between rational appeal and purchase intentions.

*H8b*: Self-referencing and self-brand congruity play a chain mediating role between emotional appeal and purchase intentions.

### 2.3. Model specification

**Figure 1:**
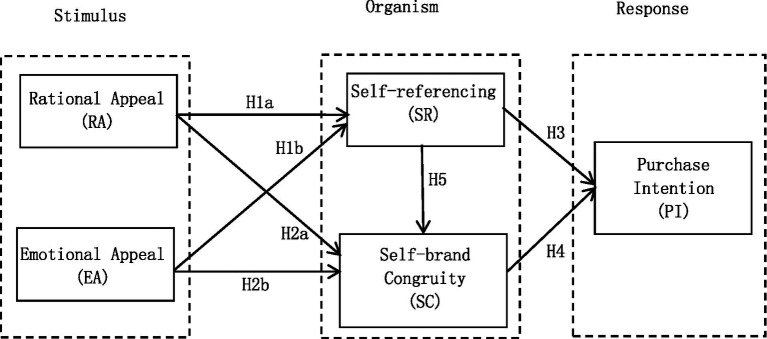
Conceptual model.

Based on the above assumptions, the research model of this paper is constructed as follows (Figure 1). In this paper, the stimulus factors are rational appeal (RA) and emotional appeal (EA), the organism state is self-referencing (SR), self-brand congruity (SC), and the behavioral response is purchase intention (PI).

## 3. Research design

### 3.1. Measures

The questionnaire consists of two parts, the first part is the basic demographic information (including gender, age, and education level, etc.). The second part is the measurement of research variables (including rational appeal, emotional appeal, self-referencing, self-brand congruity, and purchase intention). In this study, questionnaire items using measurement variables were all from existing studies, and were modified according to the live streaming e-commerce scenario to ensure the accuracy and effectiveness of measurement items. Rational appeal was measured by using Resnik and Stern (1977) scale. Emotional appeal was measured by using De Pelsmacker and Geuens (1997) scale, and provocation and eroticism were excluded from the study due to legal restrictions on live streaming. Debevec (1995) scale was used for self-referencing. Self-brand congruity was adopted from Phua and Kim (2018) scale. Purchase intention was measured using Zeithaml et al. (1996) scale. Each item of the study variables was measured using a seven-point Likert scale (ranging from 1 = “completely disagree” to 7 = “completely agree”).

To enhance the validity of the formal survey, a small preliminary survey was conducted between October 15 and 16, 2022, and 100 questionnaires were distributed. According to the results of the analysis of the pre-survey data, one measure of the independent variable emotional appeal had a factor loading (loadings) of 0.601, which was less than the standard 0.707 (Shimp and Sharma, 1987), so this questionnaire item was considered for deletion and the remaining items constituted the formal questionnaire for this study. The pre-study data were not applied to the final data analysis.

### 3.2. Data collection

The formal survey used a convenience sample for data collection, and the questionnaire was created on the Chinese online survey platform, Questionnaire Star,^1^ to a sample of Internet users in mainland China. Respondents were informed of the purpose of the study before filling out the questionnaire and were aware that the survey was anonymous. The questionnaires were distributed from October 17 to October 23, 2022.

In the formal survey, 509 questionnaires were collected, and 482 questionnaires were obtained excluding the invalid questionnaires, and the percentage of valid questionnaires was 94.70%. The gender of the respondents was 55.40% female (*N* = 267) and 44.60% male (*N* = 215). 84.00% of the total sample were aged 18–35, and nearly 77.80% of the respondents had university or higher education. According to the Discovery Report “2022 Live E-Commerce Industry Report” (Discovery Report, 2022), 18–37 years old are the main group of live e-commerce consumers. The “Tmall 618 Taobao Live Consumer Portrait” (Taobao Live, 2019) released by Chinese live e-commerce giant Taobao Live also shows that the top three main consumer groups are 22–32, 12–21, and 33–40 years old. Taken together, the demographic information distribution of the survey respondents is broadly consistent with the demographic information characteristics of the current main consumer groups of live shopping. The demographic information of the sample is shown in Table 1.

**Table 1:** Demographic characteristics of sample (*N* = 482).

Variables	Item	Frequency	Ratio (%)
Gender	Male	215	44.60%
	Female	267	55.40%
Age	Under 18	17	3.50%
	18–24	283	58.70%
	25–35	122	25.30%
	Above 35	60	12.40%
Education	Below junior high school	14	2.90%
	Junior high school	23	4.80%
	High school	32	6.60%
	Specialist	38	7.90%
	Bachelor	235	48.80%
	Master	124	25.70%
	Phd and above	16	3.30%

## 4. Results

In this study, partial least squares structural equation model (PLS-SEM) was selected for data analysis and model test. The main reasons for PLS-SEM were as follows: first, PLS-SEM method is more suitable for exploratory research, and second, it is suitable for small sample research (Hair et al., 2019). Based on this, this paper selects SmartPLS 4.0 software for analysis.

### 4.1. Mean and standard deviations

Table 2 shows the arithmetic mean, standard deviation, and the relative importance of all study variables. The arithmetic mean and the relative importance of all the variables reach the average level. Thus, considering the study sample under examination and analysis.

**Table 2:** Mean, SD, excess kurtosis, and skewness.

Variables	Mean	SD	Excess kurtosis	Skewness
RA1	5.241	1.380	0.758	−0.894
RA2	5.046	1.418	0.133	−0.694
RA3	5.137	1.352	0.371	−0.705
RA4	4.934	1.488	−0.125	−0.549
RA5	5.160	1.434	0.347	−0.787
RA6	5.178	1.435	0.416	−0.852
RA7	4.952	1.453	0.298	−0.674
RA8	5.039	1.456	0.212	−0.675
RA9	5.019	1.511	−0.083	−0.690
RA10	4.934	1.424	−0.139	−0.501
RA11	4.828	1.457	−0.272	−0.454
RA12	4.587	1.663	−0.635	−0.403
RA13	4.558	1.712	−0.730	−0.389
RA14	4.766	1.587	−0.267	−0.576
EA1	4.994	1.579	0.142	−0.780
EA2	4.795	1.691	−0.389	−0.605
EA3	4.193	1.789	−0.859	−0.168
SR1	4.166	1.895	−1.041	−0.221
SR2	4.533	1.671	−0.485	−0.498
SR3	4.577	1.731	−0.562	−0.463
SB1	4.371	1.666	−0.672	−0.310
SB2	4.394	1.657	−0.569	−0.297
PI1	4.950	1.447	−0.053	−0.597
PI2	4.732	1.560	−0.434	−0.445
PI3	4.471	1.738	−0.674	−0.391

### 4.2. Measurement model inspection

The test of measurement model is mainly to test various indicators of reliability, convergent validity, and discriminant validity (Hair et al., 2019). Reliability is an index to evaluate the stability and congruity of the model, which was evaluated by Cronbach’s Alpha coefficient and combined reliability (CR). Cronbach’s Alpha coefficient below 0.6 is considered untrustworthy, and above 0.8 indicates good reliability (Bagozzi and Yi, 1988). The minimum value of CR is 0.7, and the larger the value, the more the item can measure the latent variable (Nunnally, 1978). In this analysis, the reliability analysis results are shown in Table 3. Cronbach’s Alpha coefficients and CR coefficients of all dimensions are greater than 0.7, indicating that each item has good reliability and good internal consistency.

**Table 3:** Reliability and validity results of measurement model.

Variables	Items	Loadings	Cronbach’s alpha	Rho_A	CR	AVE
RA	RA1	0.744	0.953	0.957	0.958	0.622
	RA2	0.796				
	RA3	0.777				
	RA4	0.803				
	RA5	0.777				
	RA6	0.723				
	RA7	0.788				
	RA8	0.759				
	RA9	0.818				
	RA10	0.824				
	RA11	0.828				
	RA12	0.812				
	RA13	0.782				
	RA14	0.799				
EA	EA1	0.817	0.808	0.816	0.886	0.722
	EA2	0.873				
	EA3	0.859				
SR	SR1	0.889	0.880	0.883	0.926	0.807
	SR2	0.904				
	SR3	0.901				
SC	SC1	0.934	0.861	0.862	0.935	0.878
	SC2	0.940				
PI	PI1	0.894	0.863	0.864	0.916	0.785
	PI2	0.886				
	PI3	0.878				

Validity is an index to comprehensively evaluate whether the measurement model can accurately reflect the purpose and requirements of evaluation. Generally, it is tested from two aspects: convergence validity and discriminative validity. Convergence validity measures mean variance extraction (AVE) and factor load values. According to Bagozzi (1981), the AVE value is suggested to be greater than 0.5, and the higher the AVE, the better the convergent validity. According to the suggestion of Carmines and Zeller (1979), a loadings value greater than 0.707 has a good convergence validity. As can be seen from Table 3, the AVE and loadings values of each measurement item in this study are all greater than 0.5 and 0.707, indicating good convergent validity of the scale.

Discriminant validity refers to the distinction between items in different dimensions. In this paper, the Fornell–Larcker standard was adopted to evaluate the scale’s discriminant validity (Fornell and Larcker, 1981). If the square root of the AVE of factors is greater than all inter-construct correlations, the discriminant validity is supported. As shown in Table 4, the value on the diagonal (in bold) is the square root value of each variable AVE, which is larger than the correlation coefficient of all other variables, indicating that all dimensions of the scale used in this study have good discriminative validity.

**Table 4:** Fornell–Larcker criterion (comparison of square root of average variance extracted and inter-construct correlations).

Variables	Emotional appeal	Purchase intention	Rational appeal	Self-brand congruity	Self-referencing
Emotional appeal	**0.850**				
Purchase intention	0.337	**0.886**			
Rational appeal	0.418	0.438	**0.788**		
Self-brand congruity	0.461	0.482	0.406	**0.937**	
Self-referencing	0.472	0.459	0.396	0.639	**0.898**

The square root of average variance extracted (AVE) is shown on the diagonal of the matrix.

Discriminant validity refers to the distinction between items in different dimensions. In this paper, the Heterotrait-monotrait ratio standard was adopted to evaluate the scale’s discriminant validity, and HTMT shall be less than 0.9 (Henseler et al., 2015). As shown in Table 5, HTMT are less than 0.8, indicating that all dimensions of the scale used in this study have good discriminative validity.

**Table 5:** Discriminant validity of measurement model (HTMT).

Variables	Emotional appeal	Purchase intention	Rational appeal	Self-brand congruity	Self-referencing
Emotional appeal					
Purchase intention	0.407				
Rational appeal	0.472	0.484			
Self-brand congruity	0.548	0.559	0.439		
Self-referencing	0.555	0.526	0.425	0.730	

### 4.3. Structural model analysis

**Figure 2:**
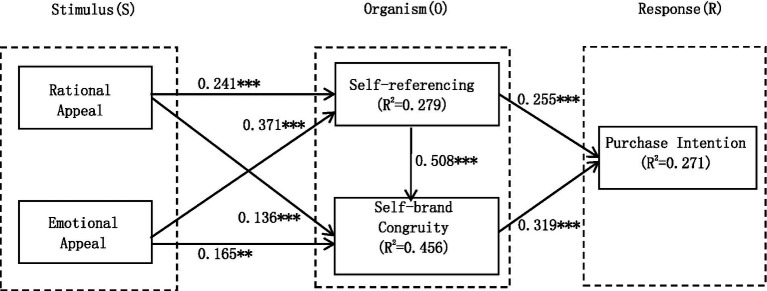
Structural equation model results diagram. ^***^p < 0.001；^**^p < 0.01.

The structural model was examined by checking the path coefficient (*β*) and coefficient of determination (*R*^2^). Figure 2 shows the results of the structural model test.

The relationship between anchor language appeals, self-referencing, self-brand congruity, and purchase intention was verified by examining the path coefficient (*β*). Rational appeal was significantly and positively correlated with self-referencing (*β* = 0.241; *p* < 0.001) and self-brand congruity (*β* = 0.136; *p* < 0.001), and hypotheses H1a and H2a held, indicating that anchors’ rational appeal had a positive influence on users’ self-referencing and self-brand congruity; emotional appeal was positively correlated with self-referencing (*β* = 0.371; *p* < 0.001) and self-brand congruity (*β* = 0.165; *p* < 0.01) were significantly positively correlated, and hypotheses H1b and H2b held, indicating that anchor emotional appeal has a positive influence on users’ self-referencing and self-brand congruity. Self-referencing was significantly and positively correlated with purchase intention (*β* = 0.255; *p* < 0.001), and self-brand congruity was significantly and positively correlated with purchase intention (*β* = 0.319; *p* < 0.001), and hypotheses H3 and H4 hold, indicating that users’ self-referencing and self-brand congruity have a positive influence on purchase intention. Self-referencing was significantly and positively correlated with self-brand congruity (*β* = 0.508; *p* < 0.001), and hypothesis H5 holds, indicating that user self-referencing has a positive effect on self-brand congruity.

As can be seen from Figure 2, the explanatory power *R*^2^ of self-referencing and self-brand congruity to purchase intention is 0.271, the explanatory power *R*^2^ of rational and emotional appeal to self-referencing is 0.279, and the explanatory power *R*^2^ of rational and emotional appeal to self-brand congruity is 0.456.

### 4.4. Intermediary effect verification

This study analyzes the mediating effect according to the method proposed by Zhao et al. (2010), and the results are shown in Table 6. Self-referencing and self-brand congruity mediate the influence of rational appeal and emotional appeal on purchase intention. The VAF values were 30.10, 31.35, 23.00, and 20.31%, respectively, indicating that self-referencing and self-brand congruity played a partial mediating role in the relationship between the two groups of independent variables and dependent variables.

**Table 6:** Results of mediation effect analysis.

Independent variables	Mediation variables	Dependent variable	Direct effects (*T* statistics)	Indirect effects (*T* statistics)	Total effects	VAF	Results
RA	SR	PI	0.144 (4.662)	0.062 (2.614)	0.206	30.10%	H6a supported
EA	SR		0.208 (7.433)	0.095 (3.380)	0.303	31.35%	H6b supported
RA	SC		0.144 (4.662)	0.043 (2.030)	0.187	23.00%	H7a supported
EA	SC		0.208 (7.433)	0.053 (2.634)	0.261	20.31%	H7b supported

VAF < 20%: no intermediary effect; 20% < VAF < 80%: partial mediation effect.

### 4.5. Verification of the effect of chain mediation

The psychological process of consumers’ purchase intention is complex, and multiple mediating variables are often needed to more clearly explain the effect of independent variables on dependent variables (MacKinnon, 2012). Through data analysis (Table 7), this study found that the influence of host language appeal on users’ purchase intention is realized through two ways: “Rational appeal—self-referencing—self-brand congruity—purchase intention” and “emotional appeal—self-referencing—self-brand congruity—purchase intention,” assuming that H8a and H8b are valid, the VAF is 21.31 and 22.39%, respectively, that is, the mediating variables self-referencing and self-brand congruity play a chain mediating role.

**Table 7:** Analysis results of chain mediation effect.

Independent variables	Mediation variables		Dependent variable	Direct effects (*T* statistics)	Indirect effects (*T* statistics)	Total effects	VAF	Results
RA	SR	SC	PI	0.144 (4.662)	0.039 (3.308)	0.183	21.31%	H8a supported
EA				0.208 (7.433)	0.060 (3.923)	0.268	22.39%	H8b supported

VAF < 20%: no intermediary effect; 20% < VAF < 80%: partial mediation effect.

## 5. Discussion

### 5.1. Research conclusions

This paper studies the influence mechanism of anchors’ language demands on users’ purchase intention in the scenario of live streaming e-commerce, as well as the role of self-referencing and self-brand congruity. After data analysis and structural equation model verification, we draw the following conclusions.

#### 5.1.1. Anchors’ language appeals have a positive impact on users’ self-referencing

The validity of hypotheses H1a and H1b indicates that the rational and emotional demands of anchors have a significant positive influence on users’ self-referencing. After receiving the information about the rational and emotional demands of anchors, users will compare these information with their own relevant experiences. Sun et al. (2019) showed that rational appeal from anchors increases consumers’ informational perceptions, triggering them to refer to factual information about the brand with their own experiences and influencing their subsequent purchase behavior, while Dong and Wang (2018) showed that emotional appeal from anchors increases the emotional connection with users, facilitating them to associate themselves with emotional information about the brand and increasing their favorability toward the brand. Increase users’ favorable perception of the brand. It is worth noting that according to the path coefficients, the path coefficient of the influence of the anchor’s language rational appeal on self-referencing is 0.241, while the path coefficient of the influence of the emotional appeal on self-referencing is 0.371, indicating that the influence of the emotional appeal is more significant than that of the rational appeal. This is consistent with research on online sales (Ahn et al., 2022). This result explains some of the impulsive consumption behavior that occurs in the broadcast room.

#### 5.1.2. Anchors’ language appeals have a positive impact on users’ self-brand congruity

The establishment of H2a and H2b holds that anchors’ rational and emotional appeal have a positive impact on users’ self-brand congruity. In live e-commerce shopping, users are influenced by the linguistic appeals of the anchor, and their perception of the brand image changes, believing that the brand image described by the anchor through language is in line with their self-image. Once the user develops this perception of convergence between self-image and brand image, a self-brand congruence effect occurs. The results showed that the path coefficients of rational appeal and emotional appeal on self-brand congruity were 0.135 and 0.165, respectively, with no significant difference. This result shows that, no matter whether the host uses a rational way or an emotional way, users will compare the information with their self-image after receiving the information. Therefore, the language appeal of anchors should adopt a combination of rationality and sensibility, so as to generate self-brand consistency among users under the joint action of both. This conclusion is consistent with the research results of Chen et al. (2022).

#### 5.1.3. Self-referencing and self-brand congruity have a positive impact on users’ purchase intentions

The validity of hypothesis H3, that self-referencing has a positive effect on users’ purchase intention, suggests that, after receiving recommendations from anchors, users will process information related to their self-concept and the resulting results will influence their consumption decisions. Consumers’ purchases are often motivated by a desire to obtain symbolic meaning for a product or service, and the self-reference effect arises when the symbolic meaning of a product is consistent with the consumer’s existing or desired sense of self (Lee and Heo, 2016; Lee and Mackert, 2017; de Graaf, 2022). Under the self-reference effect, consumers will tend to purchase products that are consistent with their self-identity or status. Therefore, anchors should strive to align the products they recommend with the user’s sense of self through verbal appeals. This is consistent with the results of Yaniv et al. (2011).

The validity of hypothesis H4, that self-brand congruity has a positive effect on users’ purchase intentions, suggests that users who receive recommendations from anchors process them in relation to their self-image, and the resulting results influence their consumption decisions. Anchors establish some connection between their recommended brands and users through language appeals. The higher the consistency between self-image and brand image of users, the higher their purchase intention will be. This result is consistent with the findings of Grénman et al. (2019) and Aw et al. (2019), which is better explains that in the practice of live streaming e-commerce, anchors constantly interact and communicate with users through language to enhance their understanding of users. The purpose is to establish a connection with the recommended products according to the self-image cognition of users, form a self-brand consistency, and then enhance the purchase intention of users.

#### 5.1.4. Self-referencing have a positive impact on self-brand congruity

The validity of hypothesis H5, that self-referencing positively influences users’ self-brand congruity, suggests that after receiving a recommendation from the anchor, users first compare it with their self-experience for referencing, then make a comparison of congruity between their self-image and brand image. Users of live e-commerce are influenced by the linguistic appeals of the anchor in watching the live broadcast, creating a self-referential effect by connecting with information that already exists. Under the self-referencing effect, the user’s self-image perception will in turn influence product (brand) preference, i.e., the user tends to choose products (brands) that match their self-image, thus building self-brand consistency. The higher this self-brand consistency is, the more likely they are to buy. This is consistent with the findings of Mehta (1999). This result tells us that in the practice of live e-commerce, the anchor should trigger the user’s self-reference through verbal appeals, forming a perception and construction of self-image and being able to associate self-image with brand image, generating self-brand congruence.

#### 5.1.5. Self-referencing and self-brand congruity mediate between language appeals and purchase intentions

The validity of hypotheses H6a and H6b, that self-referencing plays a partially mediating role between anchors’ language appeals and users’ purchase intentions, suggests that anchors recommend products to users by means of rational appeal and emotional appeal, and these messages cause users to engage in self-referential processing, and when self-referential congruity is high, users’ purchase intentions are also high. This result, is in line with the studies of Chang and Lee (2011) and Ahn et al. (2017). This result suggests that the linguistic appeal of the anchor prompts users to recall information about their selves in their memories, that they associate this information about their self-image with the product recommended by the anchor, and that they are inclined to buy the product when they find that the symbolic meaning of the product recommended by the anchor is consistent with the self-image that the consumer already has or wishes to acquire.

The validity of hypotheses H7a and H7b, that self-brand congruity partially mediates the relationship between anchors’ language appeals and users’ purchase intentions, suggests that anchors recommend products to users by means of rational and emotional appeal, and that this information causes users to engage in comparative processing of self-brand congruity, and that when congruence is high, users’ purchase intentions are also high. When users buy commodities in the broadcast room, they are not only based on quality, price and practical performance, but also whether the brand characteristics are in line with self-image as an important selection criteria (Phua and Kim, 2018; Wijnands and Gill, 2020). If users can find the consistency between the brand and self-image or evaluation from the anchor language appeal, that is, the self-brand consistency is high, consumers will buy the product. This result is in line with the studies by Sop and Kozak (2019) and Holmes (2021).

#### 5.1.6. The chain mediating role of self-referencing and self-brand congruity

Hypotheses H8a and H8b, where self-referencing and self-brand congruity play a chain mediating role between the anchor’s language appeal and the user’s purchase intention, are established. The study shows that there is a relationship in which the anchor’s language appeal triggers self-referential processing by the user, and when self-referential consistency is high, the user also makes a comparison of self-brand congruity, and when the user feels that self-brand congruity is high, his or her purchase intention is also high. This result is in line with the study of Phua and Kim (2018). The existence of chain mediation suggests a complex process from the verbal stimulation of the anchor to the generation of willingness to purchase, with a series of psychological changes that occur in between, and suggests that there is a complex processing of product information and purchase decision making behavior in live shopping.

### 5.2. Theoretical implications

1. The “black box” of consumer purchasing behavior refers to the fact that before consumers make purchases, merchants do not understand the mechanism of consumers’ purchasing behavior and purchase intention (Michon and Chebat, 2008). This study constructs a model of the mechanism of the influence of anchors’ language appeals on users’ purchase intention, which deepens our knowledge of the mechanism involved and enriches theoretical studies of consumer behavior. Revealing the “black box” consumers buy in the marketing mode of live streaming e-commerce is an innovative exploration of the black box consumers buy in live streaming e-commerce.
2. This study finds that self-referencing and self-brand congruity play an important role in promoting users’ purchase intention in the live broadcast. As Lee et al. (2020) and Yang (2018) showed, self-reference and self-brand congruence play a mediating role between external stimuli and consumers’ purchase intentions, and we further sub-found that these two factors are also important mediators of consumer roles in live e-commerce. The findings can provide theoretical references for the design of anchors’ communication discourse to users.
3. This study constructs a new research model with good explanatory power based on the SOR theoretical framework, which broadens the applicable scenarios of the SOR theoretical framework.

### 5.3. Practical significance

1. Anchors’ rational and emotional appeal are able to trigger users’ processing behavior of product information, which indicates that anchors should consider the rational and emotional appeal strategies when planning live communication discourse. The use of rational and emotional appeal strategies should be taken into account in the planning of live communication, neither can be neglected. The impact of emotional appeal is more significant. Therefore, anchors should pay emotional appeal strategies to recommend to users in the live broadcast room.
2. The study reveals the important role of self-reference effect in live marketing. The significant role of self-referencing in the model suggests that anchors should focus on stimulating consumers to produce self-referencing. On the one hand this will help promote consumers’ awareness of the product brand, on the other hand this can also promote consumers’ willingness to buy.
3. The study found that live marketing should focus on brand promotion at the same time when promoting products. The significant role of self-brand congruity in the model suggests that anchors should focus on self-brand congruity assessment of the product when designing promotion tactics, which can help enhance users’ purchase intention.

### 5.4. Limitations and future research

As with any research, this study has some limitations.

First, this study is based on convenience sampling for data collection, although this method has been widely adopted by the academic community for its convenience and low cost advantages. To draw more general conclusions, researchers may conduct surveys in different countries or regions under various situations in the future.

Second, all of the data in this study was collected by way of self-reporting, which may involve a subjective bias. Future studies should attempt to use different methods (e.g., psychological experiments, internet ethnography) and different types of data (e.g., objective data) to improve the results’ validity.

Third, we tested the mediating of self-referencing and self-brand congruity, and more work is thus needed to validate other factors which may also act as mediators.

## Data availability statement

The raw data supporting the conclusions of this article will be made available by the authors, without undue reservation.

## Ethics statement

According to national legislation and institutional requirements, this study does not require written informed consent.

## Author contributions

EM: conceptualization, funding acquisition, project administration, resources, and supervision. EM and JL: data curation, investigation, and writing—original draft. KL and JL: formal analysis and methodology. EM and KL: validation and writing—review and editing. JL: visualization. All authors contributed to the article and approved the submitted version.

## Funding

This work was supported by National Social Science Foundation Project: “Research on Advertising Algorithm Trap and its Governance” (22AXW009) and Henan University Philosophy and Social Science Application Research Major Project: “Research on Internet Platform Algorithm Governance in the Digital Economy Era” (2023-YYZD-23).

## Conflict of interest

The authors declare that the research was conducted in the absence of any commercial or financial relationships that could be construed as a potential conflict of interest.

## Publisher’s note

All claims expressed in this article are solely those of the authors and do not necessarily represent those of their affiliated organizations, or those of the publisher, the editors and the reviewers. Any product that may be evaluated in this article, or claim that may be made by its manufacturer, is not guaranteed or endorsed by the publisher.

## Footnotes

^1^ www.wjx.cn
