# Supplementary material for: Exploring the mechanism of live streaming e-commerce anchors’ language appeals on users’ purchase intention
Source: Front Psychol. 2023 Mar 9;14:1109092. doi: 10.3389/fpsyg.2023.1109092 (PMC10033604; doi:10.3389/fpsyg.2023.1109092)
Supplement: Supplementary file 1 [file Table_1.DOCX]

Supplementary Material

# Supplementary Tables

- 1. **Supplementary Tables**

**Supplementary Table 1.** **Demographic Characteristics of Sample（N=482）.**

| Variables | Item | Frequency | Ratio(%) |
| --- | --- | --- | --- |
| Gender | Male | 215 | 44.60% |
|  | Female | 267 | 55.40% |
| Age | Under 18 | 17 | 3.50% |
|  | 18-24 | 283 | 58.70% |
|  | 25-35 | 122 | 25.30% |
|  | Above 35 | 60 | 12.40% |
| Education | Below junior high school | 14 | 2.90% |
|  | Junior high school | 23 | 4.80% |
|  | High school | 32 | 6.60% |
|  | Specialist | 38 | 7.90% |
|  | Bechelor | 235 | 48.80% |
|  | Master | 124 | 25.70% |
|  | Phd and above | 16 | 3.30% |

**Supplementary Table 2.** Reliability and Validity Results of Measurement Model.

| variables | items | loadings | Cronbach's Alpha | rho_A | CR | AVE |
| --- | --- | --- | --- | --- | --- | --- |
| RA | RA1 | 0.744 | 0.953 | 0.957 | 0.958 | 0.622 |
|  | RA2 | 0.796 |  |  |  |  |
|  | RA3 | 0.777 |  |  |  |  |
|  | RA4 | 0.803 |  |  |  |  |
|  | RA5 | 0.777 |  |  |  |  |
|  | RA6 | 0.723 |  |  |  |  |
|  | RA7 | 0.788 |  |  |  |  |
|  | RA8 | 0.759 |  |  |  |  |
|  | RA9 | 0.818 |  |  |  |  |
|  | RA10 | 0.824 |  |  |  |  |
|  | RA11 | 0.828 |  |  |  |  |
|  | RA12 | 0.812 |  |  |  |  |
|  | RA13 | 0.782 |  |  |  |  |
|  | RA14 | 0.799 |  |  |  |  |
| EA | EA1 | 0.817 | 0.808 | 0.816 | 0.886 | 0.722 |
|  | EA2 | 0.873 |  |  |  |  |
|  | EA3 | 0.859 |  |  |  |  |
| SR | SR1 | 0.889 | 0.880 | 0.883 | 0.926 | 0.807 |
|  | SR2 | 0.904 |  |  |  |  |
|  | SR3 | 0.901 |  |  |  |  |
| SC | SC1 | 0.934 | 0.861 | 0.862 | 0.935 | 0.878 |
|  | SC2 | 0.940 |  |  |  |  |
| PI | PI1 | 0.894 | 0.863 | 0.864 | 0.916 | 0.785 |
|  | PI2 | 0.886 |  |  |  |  |
|  | PI3 | 0.878 |  |  |  |  |

**Supplementary Table** 3. Discriminant Validity of Measurement Model(Fornell-Larcker Criterion).

| Variables | Emotional appeal | Purchase intention | Rational appeal | Self-brand congruity | Self-referencing |
| --- | --- | --- | --- | --- | --- |
| Emotional appeal | **0.850** |  |  |  |  |
| Purchase intention | 0.337 | **0.886** |  |  |  |
| Rational appeal | 0.418 | 0.438 | **0.788** |  |  |
| Self-brand congruity | 0.461 | 0.482 | 0.406 | **0.937** |  |
| Self-referencing | 0.472 | 0.459 | 0.396 | 0.639 | **0.898** |

**Supplementary Table** 4. Results of mediation effect analysis.

| Independent variables | Mediation variables | Dependent variable | direct effects  (T statistics) | Indirect effects  (T statistics) | Total effects | VAF | Results |
| --- | --- | --- | --- | --- | --- | --- | --- |
| RA | SR | PI | 0.144(4.662) | 0.062(2.614) | 0.206 | 30.10% | H6a supported |
| EA | SR |  | 0.208(7.433) | 0.095(3.380) | 0.303 | 31.35% | H6b supported |
| RA | SC |  | 0.144(4.662) | 0.043(2.030) | 0.187 | 23.00% | H7a supported |
| EA | SC |  | 0.208(7.433) | 0.053(2.634) | 0.261 | 20.31% | H7b supported |

VAF<20%：No intermediary effect；20%<VAF<80%：Partial mediation effect。

**Supplementary Table** 5. Analysis Results of Chain Mediation Effect.

| Independent variables | Mediation variables | | Dependent variable | direct effects(T statistics) | Indirect effects(T statistics) | Total effects | VAF | Results |
| --- | --- | --- | --- | --- | --- | --- | --- | --- |
| RA | SR | SC | PI | 0.144(4.662) | 0.039(3.308) | 0.183 | 21.31% | H8a supported |
| EA |  |  |  | 0.208(7.433) | 0.060(3.923) | 0.268 | 22.39% | H8b supported |

VAF<20%：No intermediary effect；20%<VAF<80%：Partial mediation effect。
